# The clinical effects of high-frequency oscillatory ventilation in the treatment of neonatal severe meconium aspiration syndrome complicated with severe acute respiratory distress syndrome

**DOI:** 10.1186/s12887-021-03042-y

**Published:** 2021-12-10

**Authors:** Guang Yang, Yunxia Qiao, Xinxin Sun, Tiandan Yang, Aiying Lv, Min Deng

**Affiliations:** Department of Pediatrics, Shanxi Medical University, No. 56 xinjian north Road, Yingze District, Taiyuan City, 030001 Shanxi Province China

**Keywords:** high-frequency oscillatory ventilation, meconium aspiration syndrome, acute respiratory distress syndrome, neonate

## Abstract

**Objective:**

To explore the efficacy and safety of high-frequency oscillatory ventilation (HFOV) in the treatment of severe meconium aspiration syndrome (MAS) complicated with severe acute respiratory distress syndrome (ARDS).

**Methods:**

A total of 65 infants with severe MAS complicated with severe ARDS were included in the study. The clinical efficacy of treatment for the HFOV group (n = 31) and the conventional mechanical ventilation (CMV) group (n = 34) was retrospectively analysed. The partial pressure of oxygen (PaO_2_), partial pressure of carbon dioxide (PaCO_2_), PaO_2_/fraction of inspired oxygen (FiO_2_), and oxygen index values before and at 6, 12, 24, 48, and 72 h after mechanical ventilation, the mechanical ventilation time, oxygen inhalation time, incidence of complications, and outcomes of the two groups were compared.

**Results:**

At 6, 12, 24, and 48 h after mechanical ventilation, the PaO_2_ in the HFOV group was significantly higher than in the CMV group, while the PaCO_2_ in the HFOV group was significantly lower than in the CMV group (P < 0.05). At 6, 12, 24, 48, and 72 h after mechanical ventilation, PaO_2_/FiO2 in the HFOV group was significantly higher than in the CMV group, and the OI in the HFOV group was significantly lower than in the CMV group (P < 0.05). Mechanical ventilation time, oxygen inhalation time, and the incidence of air leakage were significantly lower in the HFOV than in the CMV group (P < 0.05).

**Conclusions:**

Overall, HFOV can effectively improve lung ventilation and oxygenation function, shorten ventilator treatment time, and reduce the incidence rate of air leakage for neonatal MAS, making it a safe and effective treatment option.

## Introduction

Neonatal meconium aspiration syndrome (MAS) is a type of postnatal dyspnoea resulting from the inhalation of meconium-contaminated amniotic fluid in utero or during delivery [1]. As a serious lung disease caused by meconium, MAS leads to airway obstruction and, subsequently, pulmonary surfactant (PS) inactivation, chemical pneumonia, and persistent pulmonary hypertension of the newborn (PPHN) [2].

In China, the mortality rate of infants with MAS is 7.0%–15.8% [3]. Severe MAS is often complicated by acute respiratory distress syndrome (ARDS), which leads to additional severe clinical symptoms, making treatment more difficult and increasing the fatality rate [4]. For uneven ventilation with partial alveolar collapse and excessive expansion, conventional mechanical ventilation (CMV) often fails to achieve the anticipated clinical effect.

Compared with CMV, high-frequency oscillatory ventilation (HFOV) has the advantages of low tidal volume, low airway pressure, and reduced alveolar injury; therefore, it is an ideal means of lung-protective ventilation.

Few domestic and foreign studies have been conducted on the therapeutic effects of different ventilation methods for children with severe MAS complicated with ARDS. Accordingly, this study aimed to compare the effects of the CMV and HFOV methods on infants with these conditions.

## Data and research methods

### Study subjects

Infants with severe MAS complicated with severe ARDS who were admitted to the neonatal department of our hospital between January 2018 and May 2020 and met the inclusion criteria were recruited for this retrospective study. We assessed the need for mechanical ventilation according to each patient's condition. As long as a patient’s condition met the standard for mechanical ventilation, they underwent either CMV or HFOV treatment.

#### Inclusion criteria

1) complete medical data; 2) the basis for MAS diagnosis followed the Practice of Neonatology (5^th^ ed.) guidelines [1]; 3) the subject’s condition was classified as severe MAS due to both a fraction of inspired oxygen (FiO_2_) higher than 40% and a mechanical ventilation time longer than 48 h [5]; 4) the presence of severe ARDS, diagnosed according to the Montreux standard of neonatal ARDS (2017) [6]; 5) mechanical ventilation used as the initial respiratory support; 6) gestational age was ≥37 weeks; 7) admission time was below 24 hours.

#### Exclusion criteria

1) death within 72 h of admission; 2) congenital malformation of the lungs or brain; 3) the presence of congenital complex heart disease; 4) the presence of genetic metabolic or chromosomal diseases.

According to the ventilation methods used, the participants were divided into the HFOV and CMV groups, respectively. This study was approved by the medical ethics committee of the hospital, and written informed parental consent was obtained for participants’ inclusion in the research.

### Therapeutic method

All children were mechanically ventilated with volume-targeted ventilation (8–10 ml/kg). The initial ventilation mode was CMV (Maquet Servo-i; Siemens) or HFOV (Sophie; Fritz Stephan GmbH). In the CMV group, according to the strength of spontaneous respiration, either assist–control (A/C) mode or synchronised intermittent mandatory ventilation (SIMV) was adopted.

The initial CMV parameters were set as follows: peak inspiratory pressure (PIP) = 18-25 cmH_2_O, positive end-expiratory pressure = 4~6 cmH_2_O, and FiO_2_ = 0.4~1.0.

The initial parameters of the HFOV were set as follows: mean airway pressure (MAP) = 10~15 cmH_2_O, oscillation pressure amplitude = 20~30 cmH_2_O (with the observation that visible thoracic vibration was appropriate), FiO_2_ = 0.4~1.0, and frequency = 9~15 Hz. The increase in frequency during HFOV did not proportionately increase the emission of carbon dioxide. This was due to the different resonance frequencies of infants’ bodies at different birth weights. In our groups, we initially set the frequency for infants with a birth weight <1,500 g at 15 Hz; for infants with a birth weight >1,500 g, this was set at 10–12 Hz. We then adjusted the frequency according to the monitoring results for carbon dioxide in the blood gas.

All the participants were given a PS (Curosurf; Chiesi Pharmaceutical, Italy) via a tracheal catheter within 24 h after birth. The first dose was 200 mg/kg; additional doses of 100 mg/kg could be given when necessary. For both mechanical ventilation methods, the ventilator parameters were adjusted according to the child's condition, blood gas analysis, and chest radiograph results. When FiO_2_ was <0.4, MAP was ≤8 cmH_2_O, the child's breathing was steady, blood oxygen saturation (SpO_2_) was maintained at 90%~95%, and blood gas analysis was normal, the ventilator was gradually withdrawn to nasal continuous positive airway pressure. Other treatments included anti-infection measures, shock correction (fluid rehydration, vasoactive drugs), the correction of cardiac function, maintenance of internal stability, and nutritional support. Some PPHN children with pneumothorax were treated with closed thoracic drainage and nitric oxide (NO) inhalation.

In this study, we selected high frequencies to improve minute ventilation and reduce carbon dioxide storage. A tidal volume of <8 ml/kg was defined as low tidal volume.

### Observation index

We compared the results of blood gas analysis before and at 6, 12, 24, 48, and 72 h after mechanical ventilation between the two groups and calculated the PaO_2_/FiO_2_ and the threshold value of the oxygen index ([OI] = FiO_2_ × MAP × 100/PaO_2_). The mechanical ventilation time, oxygen use time, complications, disease outcome and prognoses of the two groups were also compared.

### Statistical analysis

The SPSS Statistics 21.0 statistical software was used for statistical processing in this study. The measurement data were expressed as mean ± standard deviation (SD), and the F-test and the independent sample t-test methods were used for comparisons between the groups. The count data were expressed as rates (%), and a chi-square (χ^2^) test was employed for comparisons between the groups. A P*-*value of <0.05 was considered statistically significant.

## Results

### General information

A total of 65 children were included in this study; 24 cases were delivered in our hospital, and 41 cases were transferred from other hospitals. Among the 65 children, 39 were male and 26 were female. The gestational age range was 37~42^+2^ weeks, and the birth weight range was 2,690~4,400 g. There were 31 cases in the HFOV group and 34 in the CMV group. There were no statistically significant differences between the two groups in terms of gestational age, admission age, birth weight, delivery mode, sex composition, Apgar score or PS administration time (P > 0.05) as shown in Table 1.

**Table 1:** Comparison of general data between the two groups

	HFOV group(n=31)	CMV group(n=34)	statistics	*P*
Gestation ($\overline{x}$±s)/week	39.43±1.37	40.13±1.44	*t* = -1.99	0.051
Admission age($\overline{x}$±s)/h	3.52±3.69	3.63±3.11	*t* = -0.13	0.899
Birth weight($\overline{x}$±s)/g	3543±364.53	3616±419.03	*t* = -0.74	0.461
Caesarean [n (%)]	13(41.9)	15(44.1)	c^2^ = 0.03	0.859
Gender [boy, n (%)]	18(58.1)	21(61.8)	c^2^ = 0.09	0.761
1 minutes Apgar score[M(P_25~75_)]	4.5(1.0)	4.0(2.0)	*F* = 0.37	0.453
5 minutes Apgar score[M(P_25~75_)]	7.0(1.0)	7.0(2.0)	*F* = 0.71	0.714
Administration time of PS [M(P_25~75_)]	7.2(5.2)	8.1(3.9)	*F* = -0.10	0.922

### Comparison of the blood gas analysis and oxygenation index between the two groups

Prior to ventilation treatment, there were no significant differences in PaO_2_, PaCO_2_, PaO_2_/FiO_2_, or OI values between the two groups (P > 0.05).

At 6, 12, 24, and 48 h after mechanical ventilation was started, PaO_2_ in the HFOV group was higher than in the CMV group, and the differences were statistically significant (P < 0.05). However, there was no statistically significant difference in PaO_2_ between the two groups at 72 h (P > 0.05).

At 6, 12, 24, and 48 h after mechanical ventilation, PaCO_2_ in the HFOV group was lower compared with the CMV group, and the differences were statistically significant (P < 0.05). However, there was no statistically significant difference in PaCO_2_ between the two groups at 72 h (P > 0.05).

At 6, 12, 24, 48, and 72 h after mechanical ventilation, PaO_2_/FiO_2_ in the HFOV group was higher than in the CMV group, and the differences were statistically significant (P < 0.05).

At 6, 12, 24, 48, and 72 h after mechanical ventilation, the OI in the HFOV group was lower compared with the CMV group, and the differences were statistically significant (P < 0.05) as shown in Table 2.

**Table 2:** Comparison of blood gas analysis and oxygen indexes between HFOV group (n=31) and CMV group (n=34) ($\overline{x}$±s)

	Before treatment	6 h after treatment	12 h after treatment	24 h after treatment	48 h after treatment	72 h after treatment
PaO_2_						
HFOV group	43.30±3.33	60.61±3.30	68.32±5.12	74.35±3.06	78.37±5.22	82.77±4.11
CMV group	44.41±2.83	57.26±4.83	65.00±7.06	70.56±6.81	74.41±4.57	82.44±6.82
*t*	-1.39	3.23	2.16	2.94	3.24	0.94
*p*	0.169	<0.01	0.035	<0.01	<0.01	0.349
PaCO_2_						
HFOV group	61.03±3.81	54.03±3.77	50.42±5.20	46.97±5.60	42.74±3.68	39.97±3.38
CMV group	61.65±3.59	57.09±5.46	54.44±3.70	51.41±5.03	46.91±5.41	41.24±4.06
*t*	-0.84	-2.60	-3.57	-3.436	-3.60	-1.36
*p*	0.403	0.012	<0.01	<0.01	<0.01	0.179
PaO_2_/FiO_2_						
HFOV group	132.71±7.07	190.58±12.20	217.19±13.27	268.52±17.64	304.45±17.93	347.74±20.79
CMV group	130.32±6.41	182.21±16.46	209.47±12.65	253.71±17.82	285.91±33.49	338.03±17.44
*t*	1.43	2.34	2.40	3.36	2.82	2.05
*p*	0.158	0.022	0.019	<0.01	<0.01	0.045
OI						
HFOV group	17.82±1.62	15.52±2.43	13.28±1.65	10.07±1.45	8.00±1.97	6.25±2.66
CMV group	17.33±2.35	16.64±1.41	14.19±1.55	11.19±1.46	9.12±1.29	7.29±1.32
*t*	1	-2.24	-2.27	-3.10	-2.83	-2.03
*p*	0.321	0.025	0.026	<0.01	<0.01	0.047

2.3 Comparison of oxygen use duration and complications between the two groups

The duration of mechanical ventilation, as well as oxygen use in the HFOV group, were shorter compared with the CMV group, and the differences were statistically significant (P < 0.05).

The incidence of air leakage in the HFOV group was lower compared with the CMV group, and the difference was statistically significant (P < 0.05).

There were no statistically significant differences between the complication or cure rates for the two groups (P > 0.05) as shown in Table 3.

**Table 3:** Comparison of mechanical ventilation time, oxygen time, complications and outcomes between HFOV group (n=31) and CMV group (n=34)

	HFOV group	CMV group	statistics	*P*
Mechanical ventilation time (hours, $\overline{x}$±s)	85.57±5.30	95.62±4.39	*t* = -8.50	<0.001
Using oxygen time (hours, $\overline{x}$±s)	102.03±10.64	109.62±8.59	*t* = -3.18	<0.01
Complication [n (%)]				
Air leakage	2(6.5)	9(26.5)	χ^2^ = 4.62	0.032
PPHN	5(16.1)	7(22.6)	χ^2^ =0.41	0.52
NEC	0(0)	1(2.9)	χ^2^ =0	1
IVH≥III	2(6.5)	3(8.8)	χ^2^ =0	1
Pyemia	7(22.6)	5(14.7)	χ^2^ =0.67	0.527
Shock	6(19.4)	4(11.8)	χ^2^ =0.25	0.615
HIE	11(35.5)	9(26.5)	χ^2^ =0.62	0.432
Death	2(6.5)	1(2.9)	χ^2^ =0.01	0.935

## Discussion

The small airways of children with MAS can become partially blocked by meconium, generating a valve effect. As a result, air is not easily discharged when exhaling; as a result, the alveoli can become over-inflated, leading to localised emphysema. Once scattered atelectasis is formed, if the small airway is completely blocked by meconium atelectasis, the child will be born with severe lung ventilation dysfunction. Due to a variety of factors including inactivated PS and chemical inflammation, acute and widespread destruction of the pulmonary alveolar–capillary barrier occurs, eventually causing ARDS [7]. The CMV method is the primary clinical technique for the treatment of severe MAS. In contrast, HFOV is recommended as a salvage treatment for severe respiratory failure in neonates, using low tidal volume (tidal volume <8 ml/kg), supra-physiologically high respiratory rate, and ventilation at a low respiratory pressure to improve gas exchange [8].

The results of this study showed that at 6, 12, 24 and 48 h after the start of mechanical ventilation, PaO_2_ in the HFOV group was higher than in the CMV group, while PaCO_2_ was lower in the HFOV group than in the CMV group, and all of these differences were statistically significant. Uneven airway ventilation and decreased pulmonary compliance are important pathological changes in severe MAS cases that initially manifest as severe hypoxaemia and/or carbon dioxide retention. Pulmonary gas distribution is more uniform when HFOV is adopted; thus, balanced alveolar ventilation can be better maintained and, accordingly, has an important role in improving poor oxygenation [9]. Additionally, when exhalation is still active, HFOV can reduce the occurrence of dynamic overinflation, which is conducive to the discharge of CO_2_ and can effectively alleviate the occurrence of hypoxia and hypercapnia within a short time.

In the CMV group, the tidal volume was typically 8~10 ml/kg in the early stage of ARDS to maintain the SpO_2_ fluctuation at 90%~95%. We believe that CMV requires a higher tidal volume and higher PIP to ensure ventilation in patients compared with relatively uniform and low-lung-volume disease. However, this can increase the risk of pulmonary air leakage, which is contrary to the treatment principles of neap tidal volume and low plateau pressure for ARDS [10, 11].

In a multi-centre study of 482 adults treated with mechanical ventilation, Needham et al. [12] found that the ARDS fatality rate increased by 23% for each 1 ml/kg of initially set tidal volume and by 15% for each 1 ml/kg increase in the tidal volume set by subsequent parameters. Zimova et al. [13] found that the median tidal volume of HFOV for maintaining a normal CO_2_ level in neonatal non-interstitial pneumonia was 1.67 ml/kg. In a study of 53 preterm infants who received HFOV for neonatal RDS, Tuzud et al. [14] reported that a tidal volume >2.4 ml/kg was rarely needed to maintain normal PaCO_2_ levels. This was consistent with our finding that normal blood gas could be maintained by setting an HFOV tidal volume <2.5 ml/kg. This indicated that the neap tide ventilation strategy of HFOV was suitable for the pathophysiological changes of MAS and ARDS.

Poddutoor et al. [15] demonstrated that the application of HFOV in the emergency treatment of neonatal acute lung injury could rapidly improve oxygenation and blood gas analysis results within 2 h from the start of ventilation. In the current study, we found that PaO_2_/FiO_2_ was higher in the HFOV compared with the CMV group at 6, 12, 24, 48, and 72 h after the start of mechanical ventilation, while the OI of the HFOV group was lower compared with the CMV group at these time points. The differences were statistically significant.

High-frequency oscillatory ventilation can reopen the small airway and the alveoli that have been occluded in calse of ARDS. This will provide effective ventilation and allow air exchange at a low alveolar inflation pressure and relatively constant pressure and volume, which can improve oxygen and maintain it at a high level for an extended time.

The Canadian Paediatric Society [16] recommends PS treatment for children undergoing mechanical ventilation for MAS. In the current study, both groups were given PS treatment in the early postnatal period, and pulmonary compliance improved rapidly as a result. In addition, the more appropriate ventilation mode effectively alleviated the severity of ARDS, making the mechanical ventilation duration and oxygen use time in the HFOV group shorter.

As a special ventilation method, there remains controversy regarding the safety of the clinical application of HFOV, particularly in terms of the risk of air leakage and intracranial haemorrhage [17]. In this study, there was a lower incidence of air leakage in the HFOV group than in the CMV group, and there were no statistically significant differences in the incidence of intracranial haemorrhage and other complications between the two groups.

Overall, HFOV can reduce the extent of lung injury caused by local lung overexpansion and repeated alveolar opening and closing, optimise the ventilation effect, and reduce the occurrence of air leakage. It can also significantly alleviate PaCO_2_ issues and effectively maintain this aspect at normal levels, thus avoiding the risk of cerebral vasodilation and increased cerebral blood flow caused by hypercapnia.

When collecting the data, we found a higher incidence of intermittent hypoxemia in the CMV group, which may have contributed to the occurrence of or further deterioration of cases of PPHN. The superior pulmonary ventilation effect of HFOV could reduce intrapulmonary shunt, which was conducive to the action of NO on pulmonary vessels and improved oxygenation. Although this research was a retrospective study with a small sample size, and large-scale clinical studies are needed to further clarify the optimal application time of HFOV and its influence on the prognosis of ARDS, our findings support HFOV as a safe and effective option for the treatment of MAS.

One of the reasons why HFOV is better suitable for neonates compared with other patient groups is because a smaller body surface area results in improved lung tissue resonance and corresponding ventilation effects. Using HFOV can improve ventilation and oxygenation in patients with severe MAS [18], alleviate lung inflammatory reactions [19], effectively relieve the severity of ARDS [20] and shorten the duration of mechanical ventilation and oxygen use, thereby allowing patients to reach the remission stage quickly. Accordingly, it is more in line with the pulmonary protective ventilation strategy than CMV [21] and is worthy of clinical promotion.

There were several limitations in this study. First, the research was a retrospective study, as opposed to a randomised controlled trial; accordingly, bias was introduced. Second, based on our design, the targeted tidal volumes were 8–10 ml/kg, which may have had some influence on the efficacy of the treatments. Third, although the results regarding OI were statistically significant, few of our additional findings were clinically significant; therefore, further research on this topic is needed in the future.

## Conclusion

Overall, HFOV can effectively improve lung ventilation and oxygenation function, shorten ventilator treatment time, and reduce the incidence of air leakage for neonatal MAS, making it a safe and effective treatment method.

## Data Availability

The datesets used or analyzed during the current study are available from the corresponding author on reasonable request.
